# Human Parainfluenza Virus 3 Phosphoprotein Is a Tetramer and Shares Structural and Interaction Features with Ebola Phosphoprotein VP35

**DOI:** 10.3390/biom11111603

**Published:** 2021-10-29

**Authors:** Joaquin Rodriguez Galvan, Brianna Donner, Cat Hoang Veseley, Patrick Reardon, Heather M. Forsythe, Jesse Howe, Gretchen Fujimura, Elisar Barbar

**Affiliations:** 1 Department of Biochemistry & Biophysics, College of Science, Corvallis, OR 97331, USA; jrg644@nyu.edu (J.R.G.); donnerb@oregonstate.edu (B.D.); hoangc@oregonstate.edu (C.H.V.); forsythh@oregonstate.edu (H.M.F.); howejess@oregonstate.edu (J.H.); fujimurg@oregonstate.edu (G.F.); 2 NMR Facility, Oregon State University, Corvallis, OR 97331, USA; reardonp@oregonstate.edu

**Keywords:** HPIV3, EBOV, phosphoprotein, intrinsically disordered proteins, VP35, LC8, aggregation, host-pathogen interactions, RNA

## Abstract

The human parainfluenza virus 3 (HPIV3) poses a risk for pneumonia development in young children and immunocompromised patients. To investigate mechanisms of HPIV3 pathogenesis, we characterized the association state and host protein interactions of HPIV3 phosphoprotein (HPIV3 P), an indispensable viral polymerase cofactor. Sequence analysis and homology modeling predict that HPIV3 P possesses a long, disordered N-terminal tail (P_TAIL_) a coiled-coil multimerization domain (P_MD_), similar to the well-characterized paramyxovirus phosphoproteins from measles and Sendai viruses. Using a recombinantly expressed and purified construct of P_MD_ and P_TAIL_, we show that HPIV3 P in solution is primarily an alpha-helical tetramer that is stable up to 60 °C. Pulldown and isothermal titration calorimetry experiments revealed that HPIV3 P binds the host hub protein LC8, and turbidity experiments demonstrated a new role for LC8 in increasing the solubility of HPIV3 P in the presence of crowding agents such as RNA. For comparison, we show that the multimerization domain of the Zaire Ebola virus phosphoprotein VP35 is also a tetramer and binds LC8 but with significantly higher affinity. Comparative analysis of the domain architecture of various virus phosphoproteins in the order Mononegavirales show multiple predicted and verified LC8 binding motifs, suggesting its prevalence and importance in regulating viral phosphoprotein structures. Our work provides evidence for LC8 binding to phosphoproteins with multiple association states, either tetrameric, as in the HPIV3 and Ebola phosphoproteins shown here, or dimeric as in rabies virus phosphoprotein. Taken together the data suggest that the association states of a virus-specific phosphoprotein and the complex formed by binding of the phosphoprotein to host LC8 are important regulators of viral function.

## 1. Introduction

**Figure 1:**
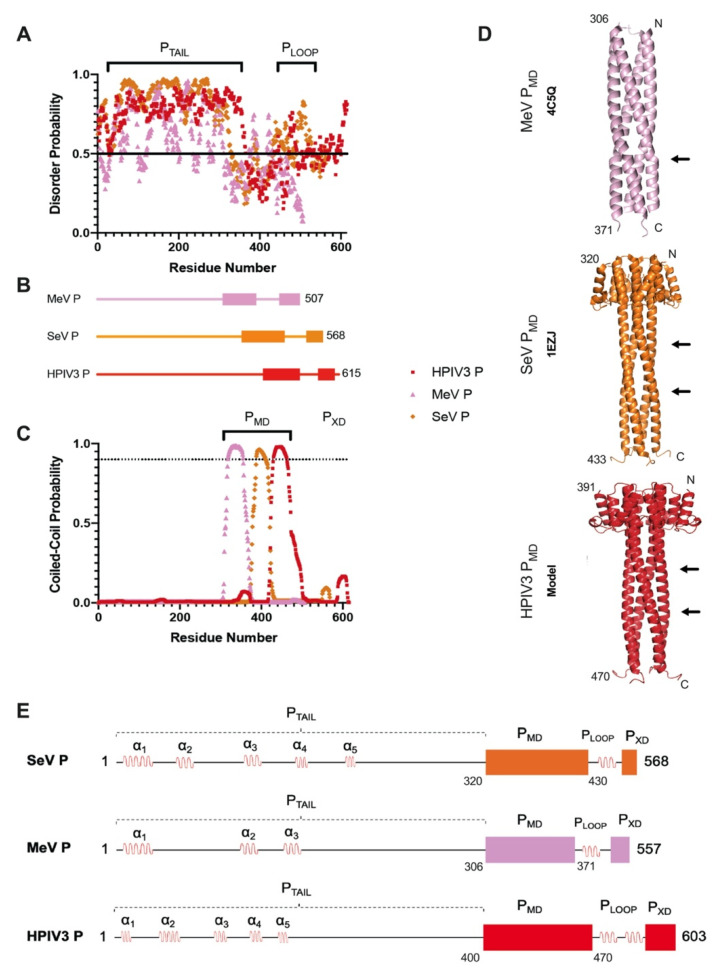
Construct design and homology P_MD_ structures based on measles and Sendai virus structural information. (**A**) Individual residue disorder probability from IUPred; IUPred scores were plotted for each residue in the query sequences. (**B**) Overall domain architecture of the different phosphoprotein. (**C**) Coiled-coil probability scores (P-score) were plotted against the residue number of the different phosphoproteins using the WaggaWagga server, showing a coiled-coil P_MD_ close to the C-terminus. (**D**) PDB structures of MeV P_MD_ (4C5Q) and SeV P_MD_ (1EZJ), HPIV3 P_MD_ was modeled using SeV P_MD_ as a template based on sequence identity. Arrows indicate coiled-coil kinks. (**E**) Detailed sequence analysis for all three viruses. Black lines, disordered regions; red loops, short alpha-helical regions; rectangles, phosphoprotein multimerization domain; squares, C-terminal domain (XD). Orange, pink, and red colors represent SeV P, MeV P, and HPIV3 P, respectively.

The human parainfluenza virus (HPIV), in the *Paramyxoviridae* family, is a cluster of seasonal respiratory viruses (RVs) that generally cause mild to moderate respiratory impairments [1]. HPIV infections typically follow a common cold-like trajectory in healthy adults but elevate the risk of pneumonia in children and immunocompromised patients [2]. HPIV variants HPIV3 and HPIV4 are associated with the poorest clinical outcomes; both are highly transmissible, particularly in hospitals, and accountable for increased ICU admission rates in children [2,3]. HPIV3, in particular, causes high morbidity yet very low mortality among vulnerable patients [4]. The underlying reasons for these observations as well as the molecular pathogenesis of HPIV3 infections are unclear. There is an urgent need to identify novel mechanisms at the host–pathogen interface; unveiling these processes will contribute substantially to effective treatment and vaccine efforts against HPIVs and hypothetical emergent paramyxoviruses. Phosphoproteins show promise as experimental systems for investigation of pathogenic pathways in mononegaviruses, because they are indispensable for many processes in viral replication and host–pathogen interactions. Although the multifunctional polymerase cofactor phosphoprotein P gene is found in every mononegavirus, its length, domain architecture, and sequence identity, vary by family [5]. HPIV3 P is a 603-residue, highly disordered protein (Figure 1) consisting of a long N-terminal intrinsically disordered tail (P_TAIL_) with interspaced alpha helices (α_1–5_), a multimerization domain (P_MD_), a linking loop (P_LOOP_), and a C-terminal helical domain (P_XD_) [6,7,8].

The multifunctionality of HPIV3 P is attributed to its structural disorder, phosphorylation of P_TAIL_, association state, and protein–protein interactions [9,10,11]. The oligomerization state of HPIV3 P was heretofore unknown, while phosphoproteins from other paramyxoviruses were controversially reported as trimers and tetramers [5,12,13]. Further, coexisting P oligomerization states are observed in paramyxo- and broader mononegaviruses, suggesting the existence of multiple association states [12,14,15,16,17]. Structural analysis of the paramyxoviral P_MD_ shows that a coiled-coil kink allows for a more dynamic P structure, likely facilitating a wide variety of conformations required for association with multiple binding partners [14]. Phosphoprotein interactions with N and L have been functionally characterized in HPIV3 and other paramyxoviruses; they are indispensable for RNA synthesis, RNA packaging, and IB formation [7,18,19,20]. Nonetheless, few data are available on HPIV3 P interactions with host proteins.

A protein similar to HPIV3 P is the Zaire Ebolavirus (EBOV) VP35 phosphoprotein. EBOV also belongs to the order Mononegavirales and the Filoviridae family [1]. EBOV infections can have up to 90% mortality due to the lack of effective therapeutics and vaccines [21,22]. Similar to HPIV3 P, EBOV VP35 is an oligomeric polymerase cofactor with additional functions such as innate immune antagonism [23]. EBOV VP35 is reported to exist as either a dimer, trimer, or tetramer [17,24,25,26,27] and, thus, like HPIV3 P, its high level of disorder and propensity to aggregate hindered biophysical characterization.

Using sequence analysis tools and our recently reported algorithm, LC8Pred, we identified a potential LC8 binding motif within the P_TAIL_ domain of HPIV3 P (Figure 1B) and also in EBOV VP35. LC8, also referred to as DYNLL1 in humans, is a small globular protein first discovered as part of the dynein motor protein complex [28]. This 20 kDa protein stabilizes the disordered region of the dynein intermediate chain IC74 by promoting self-association, thus securing the link between the motor heads and the cargo attachment complex [29]. Importantly, loss-of-function studies deem LC8 essential for eukaryotic viability [30]. Growing evidence shows LC8 is indispensable for numerous cellular processes beyond dynein-associated intracellular trafficking, such as neurotransmission, cell division, autophagy, apoptosis, nuclear transport, gene regulation, and others; there are over 100 LC8 binding partners [31,32]. LC8 functions mainly as structural support, holding two intrinsically disordered regions of its binding partners by binding their specific LC8 anchor motifs [31]. Anchor motifs are more diverse in sequence than the initially identified KXTQTX motif; however, features, such as residue volume, polarity, position, and flexibility within the anchor and surrounding residues, are conserved among binding clients [33,34].

Interestingly, LC8 is reported to be hijacked by viruses of different phyla [35]. For example, with the rabies virus phosphoprotein (RaV P), the interaction was confirmed both in vitro and in neuronal models of rabies virus infection [36,37]. LC8 association appears to be essential for stabilizing a conformation of RaV P that is indispensable for viral RNA synthesis [26]. Although VP35 is structurally different from RaV P, its interaction with LC8 is likely associated with functional regulation in a similar way [38]. While most models postulate LC8 binding as a way of hijacking the dynein complex for retrograde intracellular trafficking [35], there is ample evidence that LC8 plays more of a structural role [39,40]. Importantly, only a handful of viral phosphoproteins are known to interact with LC8 [24,36], indicating that a complex evolutionary process, not yet well defined, may be critical for comprehension of virus-specific mechanisms.

Here, we report extensive biochemical characterization of the interaction between LC8 and HPIV3 P. We also show that the multimerization domain is a tetramer, an associate state that is unusual for LC8 binding partners since they are primarily dimers. The tetrameric association state is not unique to HPIV3 P; we here show that EBOV VP35 is also a tetramer. We propose that both LC8 binding and the type of association states are important in regulating LC8 binding viruses.

## 2. Materials and Methods

### 2.1. Construct Design and Protein Purification

P_MDL_ constructs containing a short P_TAIL_ region (residues 263–300) and P_MD_ (residues 399–470) were produced using PCR and cloned into a pET24d expression vector with an N-terminal hexa-histidine (6 × His) tag and tobacco etch virus protease (TEV) cleavage site. An EBOV VP35 construct corresponding to residues 65–145 (a kind gift from Sonia Longhi) was designed to include a short, disordered stretch containing the LC8 anchor (residues 65–82) and the multimerization domain (residues 83–145) and then cloned into a pET24d expression vector. DNA sequences were verified via automated sequencing. The recombinant vectors were then transformed into *Escherichia coli* Rosetta DE3 cells for protein expression and grown using standard LB media or autoinducing media. For LB culture, 1 mM IPTG was used to induce once growth reached an OD600 of 0.6 and growth continued at 37 °C for 4 h. For autoinduction, the cells were allowed to grow at 37 °C for 24 h. The cultures were pelleted and resuspended in 50 mM phosphate buffer with 300 mM NaCl, 10 mM imidazole, and 1 mM NaN3 at pH 8 and incubated on ice. After sonication and centrifugation, the cells were purified under native conditions by affinity chromatography using TALON His-Tag resin with an elution buffer containing 350 mM imidazole at pH 8. Purification was verified by SDS-PAGE. Final purification using size exclusion chromatography and a Superdex 75 provided approximately 95% pure protein.

### 2.2. Predictions Disorder, Secondary Structure, and Coiled Coil

Disorder predictions were performed using an IUPred server; score values were plotted for each residue in the query sequences of MeV P, SeV P, and HPIV3 P. Coiled-coil predictions were performed using the WaggaWagga server. Coiled-coil probable regions were present in the 62nd, 65th, and 68th percentile for MeV P, Sev P, and HPIV3 P, respectively. Secondary structure predictions were performed using the PSIPRED server. All predictions were employed to construct final domain maps for each protein. Homology models were constructed using the SWISS-MODEL homology modeling server [41,42,43]. MeV P and SeV P tetramer structures were obtained from the PDB as 4C5Q and 1EZJ, respectively [44,45]. HPIV3 P was modeled using SeV P (1EZJ) as a template.

### 2.3. Pulldown Assays

Recombinantly expressed LC8 and P_MDL_ were purified on a nickel TALON affinity column via an N-terminal hexa-histidine (6 × His) tag. The 6 × His tag was cleaved from LC8 with TEV protease but was uncleaved in P_MDL_. Both proteins were then individually further purified by size exclusion chromatography (SEC). The SEC-purified proteins were then incubated with TALON nickel resin. Proteins were incubated separately (LC8 ctrl., P_MDL_ ctrl.) or combined (Pulldown) with TALON resin for two hours at 4 °C. Successive washing steps were performed using wash buffer (20 mM imidazole, 300 mM NaCl, 50 mM NaP, and 1 mM NaN3 at pH 7.5). Elution steps were conducted using elution buffer (500 mM imidazole, 300 mM NaCl, 50 mM NaP, and 1 mM NaN3 at pH 7.5).

### 2.4. Circular Dichroism

Spectra were recorded on a JASCO 720 spectropolarimeter using a 0.1 cm cell. Protein samples were dialyzed overnight in 2 L of 20 mM sodium phosphate, 100 mM NaF, and 1 mM sodium azide at pH 7.5 prior to data collection. Spectra were collected at 25 °C at a P_MDL_ concentration of approximately 20 or 40 μM. Blank (buffer) spectra were subtracted from the sample spectra after collection. For the melting curves, both 20 and 40 μM concentration P_MDL_ were scanned 10 times for each temperature point.

### 2.5. ITC

Samples were co-dialyzed overnight into 150 mM NaCl, 50 mM NaP, and 1 mM NaN3 at pH 7.5. For P_MDL_, 450 μM LC8 in a syringe was titrated into 40 μM P_MDL_ in the cell at 25 °C. For EBOV VP35 65–145, 195 μM of LC8 in a syringe was titrated into 15 μM EBOV VP35 65–145 in the cell. A binding thermogram was obtained with a VP-ITC microcalorimeter (Microcal, Westborough, MA, USA).

### 2.6. Analytical Ultracentrifugation

LC8 and P_MDL_ samples were dialyzed into a 4 L non-imidazole buffer containing 150 mM NaCl, 50 mM NaP, and 1 mM NaN3 at pH 7.5. The hexahistidine tag on P_MDL_ was cleaved, while LC8 remained uncleaved. Concentrations of each sample were 60 μM for LC8 alone, 50 μM LC8 and 90 μM P_MDL_ for a 0.5:1 ratio, 100 μM LC8 and 90 μM P_MDL_ for a 1:1 ratio, and 200 μM LC8 and 90 μM P_MDL_ for a 2:1 ratio. Samples of the complex were incubated by rocking at 4 °C overnight. Analytical ultracentrifugation (AUC) experiments were performed using a Beckman Coulter Optima XL-A ultracentrifuge equipped with absorbance optics (Brea, CA, USA). Protein-partial-specific volumes as well as buffer densities and viscosities were estimated using the software Sednterp [46]. For sedimentation velocity AUC (SV-AUC) experiments, samples were loaded into epon-2-channel sectored cells with a 12 mm optical pathlength. SV-AUC experiments were conducted at 42,000 rpm in a four-cell Beckman Coulter AN 60-Ti rotor and at 20 °C. Scans were performed continuously for a total of 300 scans per cell. Data were fit to a continuous c(S) distribution using the software SEDfit [47]. Sedimentation coefficients were expressed in Svedbergs (S).

### 2.7. RNA Production

For turbidity assays, total RNA was purified from mice skin fibroblasts following the RNeasy kit (Qiagen, Valencia, CA, USA) and stored in the freezer at −80 until use [48].

### 2.8. Multi-Angle Light Scattering

Size exclusion chromatography in line with multi-angle light scattering (SEC-MALS) was carried out using a Superdex^TM^ 200 gel filtration column on an AKTA-FPLC (GE Healthcare), a DAWN multiple-angle light scattering, and an Optilab refractive index system (Wyatt Technology). Data for P_MDL_ were collected by injecting 43 μM and 89 μM protein in 150 mM NaCl and 50 mM NaP at pH 7.5 buffer. Data for VP35 65–145 were collected by injecting 100 µM protein in 150 mM NaCl and 50 mM NaP at pH 7.5 buffer. The molar mass and error analysis were determined with ASTRA software package v9, employing a Zimm light scattering model (WYATT Technology, Santa Barbara, CA, USA).

### 2.9. Turbidity Assays

Purified P_MDL_ solutions were dialyzed overnight into 30 mM NaCl and 50 mM NaP at pH 6.5, and samples were serially diluted to reach desired concentrations. Turbidity measurements were carried in Greiner bio-one UV-STAR UV-VIS 96-well plates. Twenty microliters of samples were plated in replicates of 3. Plates were then shaken for 30 s linearly at standard speed and turbidity was subsequently measured at 340 nm using a BioTek^®^ Synergy HT plate reader. Data points were collected and processed using Gen5 software, version 2.09. Kinetic turbidity measurements were performed by the same plate reader using the kinetic feature on Gen5 with 10 interspaced measurements every 2:30 min. RNA was added to a final concentration of 90 ng/μL.

### 2.10. Sequence Alignment and Phylogenetics

Viral phosphoprotein sequences from the families Paramyxoviridae, Rhabdoviridae, and Filoviridae were aligned in UniprotKB in the clustalw2 format. Genes were found under the names P, P/V, P/V/D, or VP35. Resulting alignments were used to generate a phylogenetic tree using IcyTree server [49]. In addition, all sequences were run through LC8Pred [33] to identify and highlight putative binders.

## 3. Results

### 3.1. HPIV3 P Architecture Is Similar to Phosphoproteins from Other Paramyxoviruses

Sequence analysis of HPIV3 P predicted long, disordered, and coiled-coil regions. IUPred disorder predictions on measles and Sendai viruses are shown here for comparison, as there is significantly more structural data available on their domain organization (Figure 1A). In addition to a long, disordered N-terminal domain (P_TAIL_), there was a short stretch of residues (472–488, for HPIV3 P) that fell above the disorder threshold. Previous studies on MeVP show that this region forms a small loop (P_LOOP_) between predominantly ordered domains (Figure 1A,B). WaggaWagga coiled-coil prediction outputs implied a similar trend for the three phosphoproteins, with a predicted coiled-coil starting at similar regions for MeV P, SeV P, and HPIV3 P, respectively (Figure 1C).

In addition to domain architecture, crystal structures of tetrameric MeV P_MD_ and SeV P_MD_ revealed a major kink on the coiled-coil P_MD_ structure which is thought to enhance RNA synthesis via dynamic interactions with N and L [14]. HPIV3 P_MD_ was modeled by homology utilizing the SWISS-MODEL server [41,42,50,51,52,53,54] by using the SeV P_MD_ structure (1EZJ) as a template (Figure 1D). Notably, HPIV3 P has not been shown to be tetrameric. These results combined with secondary structure predictions (data not shown) allowed us to create a detailed domain map of HPIV3 P (Figure 1E) which guided construct design for the experiments in this work. 

### 3.2. The HPIV3 P Multimerization Domain Is a Stable Coiled-Coil Tetramer

**Figure 2:**
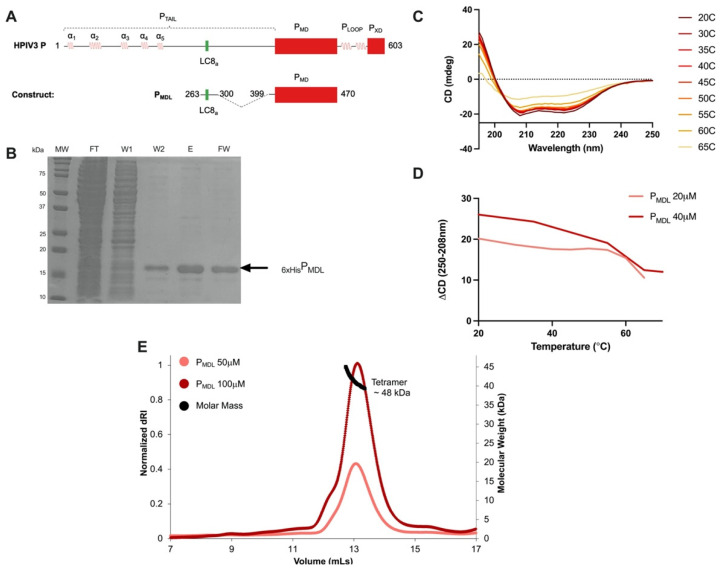
HPIV3 P forms a stable coiled-coil that is primarily a tetramer in solution. (**A**) Domain organization and construct design of P_MDL_ based on the coiled-coil predicted region in HPIV3 P. (**B**) Purification of P_MDL_ using TALON affinity nickel resin and a hexa-histidine N-terminal tag on P_MDL_. MW, molecular weight; FT, flow-through; W1, wash 1; W2, wash 2; E, elution in 350 mM imidazole; FW, final wash in 350 mM imidazole. (**C**) Circular dichroism spectra of 20 μM P_MDL_ at increasing temperature. (**D**) 40 μM P_MDL_ (two-fold concentrated) CD spectra were collected at increasing temperatures, and the 208–250 nm difference was plotted against increasing temperature points for both high and low concentrations. (**E**) Multi-angle light scattering of P_MDL_ at both 50 and 100 μM concentrations; dRI, refractive index.

To illuminate the structural features and interactions of HPIV3 P, we designed P_MDL_, a 14 kDa recombinant construct that included a short stretch of P_TAIL_, which contained a predicted LC8 binding motif and the entire P_MD_ sequence (Figure 2A). Fundamental but unanswered questions about the association state and coiled-coil nature of P_MD_ can be examined through P_MDL_. Robust expression of the construct in BL-21 *E. coli* was followed by metal affinity purification (Figure 2B). Circular dichroism (CD) spectra revealed two minima at 208 and 222 nm, consistent with a predominantly alpha-helical structure, strongly suggesting that P_MD_ is indeed a coiled-coil (Figure 2A). In thermal denaturation experiments, substantial loss of alpha helicity occurred between 55 and 60 °C (Figure 2C). A repetition of the melting curve experiment using double concentrated P_MDL_ showed no change in the determined temperature where substantial loss of alpha helicity was detected. Looking at the CD difference of the first alpha-helix minimum (250–208 nm) against temperature shows that the loss of alpha helicity occurred at similar temperatures for both 20 and 40 μM stocks, suggesting that the coiled-coil in P_MDL_ was thermostable (Figure 2D). The oligomerization state of P_MDL_, determined by SEC-MALS, was tetramer evidenced as a dominant peak with a mass of 48 kDa (Figure 2E), although a minor population corresponding to a trimer cannot be ruled out. A predominantly tetramer structure for HPIV3 P adds to the oligomeric diversity and complexity of phosphoproteins already reported in Mononegavirales. 

### 3.3. The P_TAIL_ Contains an LC8 Binding Motif That Binds LC8 In Vitro

**Figure 3:**
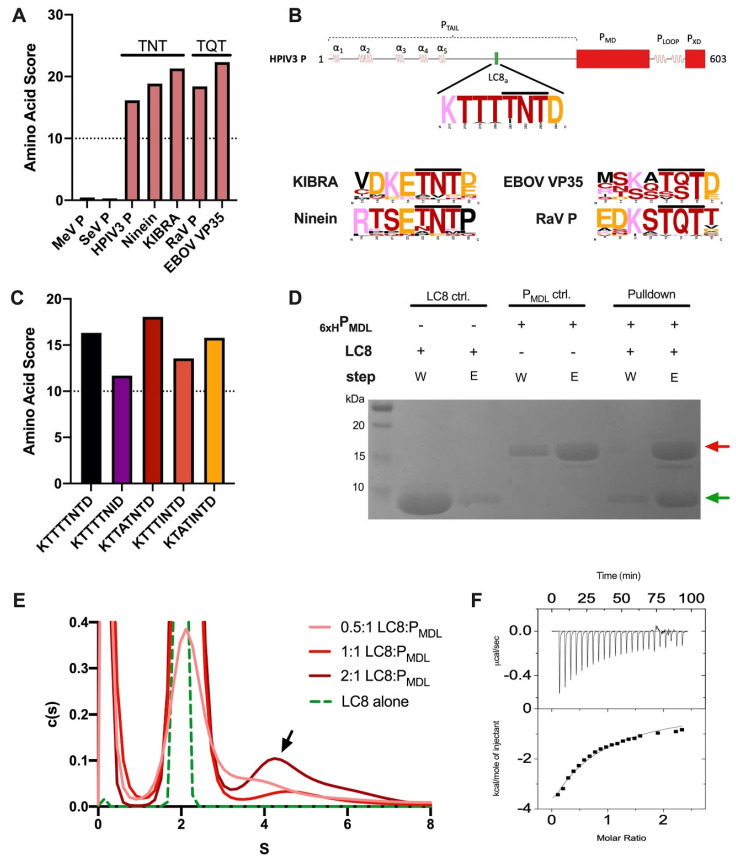
HPIV3 P contained an active LC8 binding anchor and could form higher-order oligomeric complexes. (**A**) LC8Pred algorithm prediction scores for MeV P, SeV P, HPIV3 P, and RaV P (a known LC8 binder). (**B**) Scheme of the LC8 anchor sequence location within the HPIV3 P domain map and sequence logo indicating conservation of the binding motif. (**C**) LC8Pred algorithm prediction scores for LC8 anchor motifs found at the same position in different HPIV3 P sequences reported in the UniProt database. (**D**) SDS-PAGE showing pulldown of recombinant, purified, untagged LC8 and 6 × His-tagged P_MDL_ using nickel resin. W, wash; E, elution; red arrow, P_MDL_; green arrow, LC8. (**E**) Analytical ultracentrifugation of LC8:P_MDL_ at increasing concentrations of LC8, measured at 280 nm. P_MDL_ had an impaired ability to absorb at 280 nm; the signal came from LC8 only. (**F**) ITC interaction between P_MDL_ and LC8 at 25 °C. 40 μM of P_MDL_ in the calorimeter cell was titrated with 20 injections of 500 μM LC8.

Mononegavirales phosphoproteins coordinate viral replication, transcription, and antagonize innate immune pathways [7,14]. It is therefore very likely that they use their high level of intrinsic disorder to target numerous host proteins for transient interactions. Since other Mononegaviruses, such as rabies and Ebola, have phosphoproteins that strongly interact with human LC8 [24,26], we investigated whether HPIV3 P has an LC8 recognition motif using LC8Pred [33]. Within the LC8 recognition motif, three residues act as an anchor and insert into the LC8 dimer binding cleft, namely, those at positions −1, 0, and +1; the “canonical” motif at these positions is TQT [31]. LC8Pred analysis revealed that HPIV3 P had a high-scoring motif in contrast to MeV P and SeV P which did not give above-threshold scores (Figure 3A). In addition, HPIV3 P had a “non-canonical” LC8 binding TNT motif at anchor positions. Other TNT motifs have been experimentally confirmed as human LC8 binders such as KIBRA and Ninein [33]. Comparing the LC8Pred output amino acid scores of the TNT and Mononegaviral TQT anchors revealed that HPIV3 P and the confirmed TQT and TNT binders scored well above threshold.

Next, we examined conservation within these binding motifs using clustalw2 sequence alignments. The HPIV3 P anchor is mostly conserved with minor variations at the −1 and 1 positions; TQT and TNT confirmed controls had similar conservation features (Figure 3B). In addition, individual HPIV3 P anchor sequence variants also scored above the LC8Pred threshold, suggesting that although there was minor variation, LC8 binding ability could be conserved (Figure 3C).

To confirm whether LC8 indeed interacts with HPIV3 P, we performed experiments using 6 × His-tagged P_MDL_ and demonstrated its ability to pulldown untagged LC8 by SDS-PAGE analysis as shown by its presence in the elution fraction (Figure 3D). We also verified binding by analytical ultracentrifugation sedimentation velocity experiments (AUC-SV); we mixed P_MDL_ with increasing concentrations of LC8 (0.5:1, 1:1, and 2:1 LC8:P_MDL_) and monitored the signal from LC8 only, as untagged P_MDL_ has no absorbance at 280 nm. We detected a complex with an S value of 4.2, and a tendency to form higher-order complexes upon increasing LC8 concentration (Figure 3E). Finally, isothermal titration calorimetry (ITC) gave a weak binding with an apparent kD of approximately 50 μM (Figure 3F).

### 3.4. P_MDL_ Underwent RNA-Induced Aggregation but Was Abrogated by LC8 In Vitro

**Figure 4:**
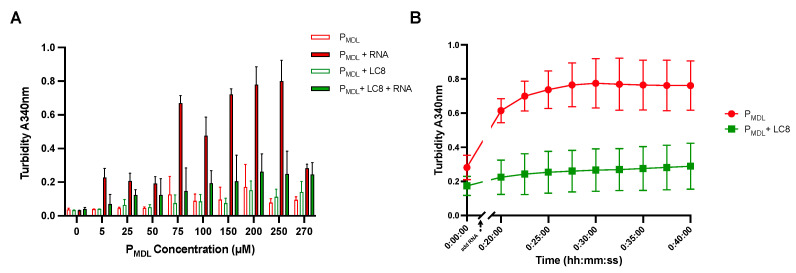
P_MDL_ aggregated upon addition of RNA as a crowding agent and LC8 antagonized aggregation in vitro. (**A**) Turbidity measurements at 340 nm at increasing concentrations of P_MDL_, P_MDL_ + RNA, P_MDL_ + LC8, and P_MDL_ + LC8 + RNA. Measurements were performed in triplicates, bars represent the mean, and error bars represent the positive standard deviation. (**B**) Kinetics of turbidity increased upon addition of RNA (time point, black arrow) in both P_MDL_ alone and P_MDL_ preincubated with LC8 in the time range of 40 min.

To investigate the role of this interaction in HPIV3 infection, we examined the possibility of LC8 regulating P aggregation. Like other disordered proteins that form soluble or insoluble aggregates [55], HPIV3 can form soluble, RNA-rich inclusion body condensates in infected cells [56,57]. To mimic the environment in the cell, we tested the ability of P_MDL_ to undergo aggregation using RNA as a crowding agent. RNA-mediated aggregation was measured by the turbidity of solutions at 340 nm; there was a significant increase in turbidity in RNA-containing solution (Figure 4A). This RNA-induced turbidity was significantly lower in P_MDL_ samples preincubated with LC8, suggesting that LC8 might hinder aggregation of HPIV3 P (Figure 4A). To probe the dynamics of aggregation, we performed time series turbidity measurements upon the addition of RNA to P_MDL_ or to P_MDL_ + LC8 preincubated solution. We observed that LC8 incubation limited the rate of turbidity increase and saturated at a lower point (Figure 4B). These results indicate that LC8 was able to regulate P_MDL_ aggregation in vitro and could play a role in regulating inclusion body formation in cells infected with HPIV3.

### 3.5. Association State of the Multimerization Domain of Ebola Zaire Phosphoprotein VP35

To compare our P_MDL_ results to other known LC8 binding phosphoproteins from the Mononegavirales order, we further investigated the Zaire Ebolavirus (EBOV) phosphoprotein VP35, a protein previously shown to bind human LC8 around position 74 where it contains a canonical TQT LC8 anchor motif (Figure 3A) [24]. In addition, VP35 has previously been reported as both a tetramer and a trimer; therefore, it remains controversial which oligomerization state is predominant with and without LC8 [17].

**Figure 5:**
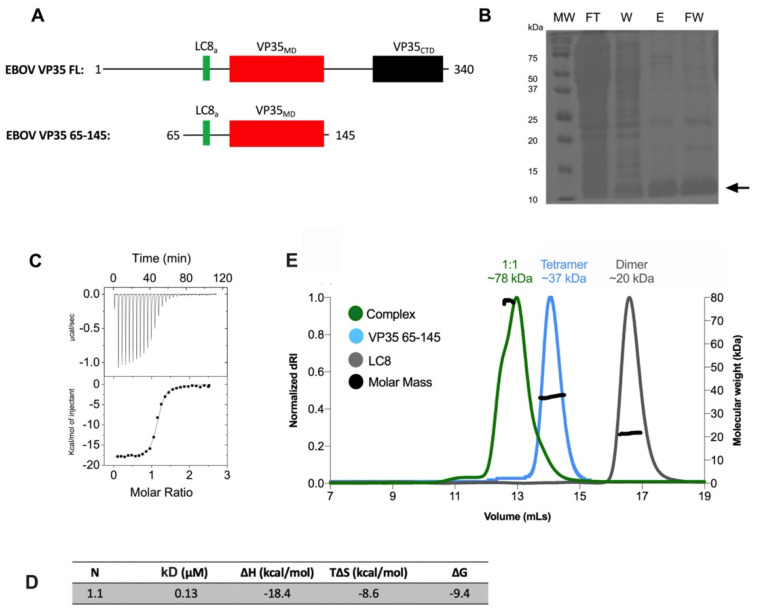
Zaire Ebolavirus phosphoprotein VP35 construct associated stronger to LC8 and had a similar oligomerization state to P_MDL_. (**A**) Domain organization and construct design of VP35 and VP35 65–145, respectively. Black lines, disordered chains; red rectangles, VP35 multimerization domain; black rectangles, VP35 C-terminal domain; green rectangles, LC8 binding anchor (LC8_a_). (**B**) Purification of VP35 65–145 using TALON affinity nickel resin and a hexa-histidine N-terminal tag on P_MDL_. MW, molecular weight; FT, flow-through; W, wash; E, elution in 350 mM imidazole; FW, final wash in 350 mM imidazole. (**C**) Isothermal titration calorimetry analysis of VP35 and LC8 interaction at 25 °C. 15 μM of VP35 in the calorimeter cell was titrated with 20 injections of 195 μM LC8. (**D**) ITC parameters from the best generated fit. (**E**) Multi-angle light scattering of VP35 45–165, LC8, and the complex at 100 μM and a 1:1 ratio, respectively. dRI, refractive index.

We designed a VP35 65–145 construct that contained the LC8 anchor (LC8_a_) within a short, disordered chain and the subsequent multimerization domain (VP35_MD_) (Figure 5A). This construct, similar to P_MDL_, can inform about the binding strength to LC8 and the oligomerization state. We then cloned, recombinantly expressed, and purified this construct for further analyses (Figure 5B). ITC showed a very strong binding affinity of VP35 65–145 to LC8 (Figure 5C,D) with a kD of 0.13 μM. SEC-MALS analysis showed that VP35 65–145 was predominantly a tetramer in solution (Figure 5E). In addition to oligomerization of the free protein, given the higher affinity of binding, we analyzed the mass of the VP35:LC8 complex. The molecular masses obtained were suggestive of a predominant 1:1 complex. Namely, 2 LC8 dimers per VP35 65–145 tetramer.

## 4. Discussion

**Figure 6:**
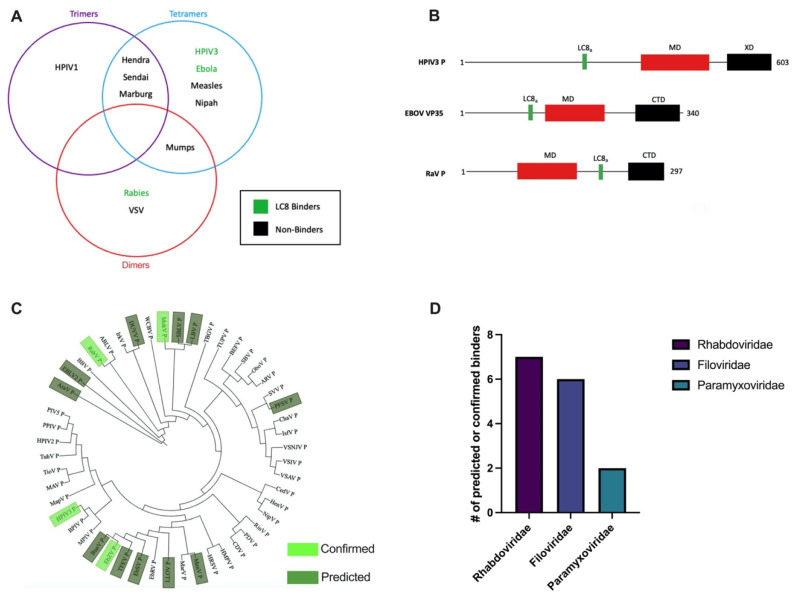
Oligomerization states and LC8 binding predictions among related viruses. (**A**) Venn diagram of phosphoprotein oligomerization states reported in the literature and those shown by our experiments [5,6,12,14,15,16,17,45,61,62,63]. Green, known LC8 binders; black, non-binders. (**B**) Domain architecture of HPIV3 P, EBOV VP35, and RaV P. Black lines, intrinsically disordered regions; red rectangles, multimerization domains; black rectangles, C-terminal domains; green rectangles, LC8 anchors. (**C**) Independent sequence alignment of phosphoprotein sequences of Filoviridae, Paramyxoviridae, and Rhabdoviridae and analysis with LC8Pred to determine binders; virus abbreviations found in Appendix A, Table A1. (**D**) Number of predicted or confirmed LC8 binding phosphoproteins in the corresponding families as shown in (**C**).

We showed that an essential, multifunctional HPIV polymerase cofactor, phosphoprotein HPIV3 P, is a thermostable, primarily tetrameric coiled-coil. Our study also demonstrates that among paramyxoviruses there are different modes of phosphoprotein-LC8 interactions, and that associated mechanisms may diverge even within closely related paramyxovirus species. Our findings underscore the importance of experimentally determining virus-specific molecular mechanisms rather than assuming similar behavior based on homology among closely related species. In good agreement with our work, HPIV3 P has been recently reported to be tetrameric by Cryo-EM [58]. Further, even though they are closely related to other paramyxoviral phosphoproteins, such as SeV P [12], the phosphoproteins reported here differ in association states [12,46] (Figure 6A). Another related example of discrepancy between reported oligomerization states is EBOV VP35. While previous literature reported dimeric, trimeric, and tetrameric structures [17,24,27], our VP35 65–145 behaved as a stable tetramer in solution, suggesting common functions with HPIV3 P, rather than with RaV P, which is a well-characterized dimer [26]. 

In addition to the oligomerization state, we also employed diverse assays to predict and confirm a novel interaction between HPIV3 P and human LC8. Although LC8 is often associated with binding dimers [31], both HPIV3 P and EBOV VP35 are tetramers independently of added LC8. Rabies virus phosphoprotein RaV P is a strong dimer in solution and does not require LC8 to promote its dimerization, but rather LC8 binding restricts the conformational space of the intrinsically disordered region and promotes a more restricted conformation that is more active [26]. Our work also revealed that LC8 might regulate P crowding and subsequent inclusion body formation, which in HPIV3-infected cells is a mechanism of enhancement of viral replication and hijacking of host machinery [59]. Our turbidity assays suggest a novel role for LC8 in regulating inclusion body formation, which in certain viruses is an essential step of their life cycle [55].

Further, we identified the LC8 binding sites in three relevant Mononegavirales families, Rhabdo-, Filo-, and Paramyxoviridae, using our predictive tool LC8Pred [33]. Sequence alignment of the corresponding phosphoproteins indicated that predicted and confirmed LC8 binders occur in related phyla forming clusters or can emerge independently (Figure 6C), suggesting that the acquisition of an LC8 recognition motif is selected in both convergent and divergent evolution. We found the most predicted and confirmed LC8 sites in Rhabdoviridae and Filoviridae compared to Paramyxoviridae (Figure 6D). Since many related viruses do not possess an LC8 site, it is important to note both the species-specific aspect of mechanisms in viral pathogenesis and the various functional roles of LC8 in different viral families.

We speculate that the limited number of phosphoprotein–LC8 interactions within these families and their diverse interaction strengths is indicative of different functions during viral infection. As a hub protein, LC8 is highly multifunctional and forms various diverse types of structural ensembles [31]. Furthermore, the examined viruses have very different tropisms [60]. Thus, they may infect cells with a high variation in biomolecular content and organization; it is therefore plausible to expect compensatory mechanisms for the absence of an interaction with LC8 in addition to the inherent variability in virus life cycles and RNA synthesis machinery.

## 5. Summary and Conclusions

Phosphoproteins are a key component of the RNA synthesis machinery of negative-sense, single-stranded RNA viruses. In this study, we aimed to address specific gaps in the literature regarding HPIV3 P and EBOV VP35, two phosphoproteins that are from different genera but even so are similar in their binding to host protein LC8. We unveiled here their oligomerization states and characterized their interaction with LC8.

Our results indicate that both the HPIV3 P and EBOV VP35 multimerization domains behaved as tetramers in solution. In addition, we show that these proteins interact with LC8 with widely different affinities, yet both formed higher-order complexes. Turbidity experiments showed that LC8 can regulate RNA-induced aggregation of HPIV3 P, suggesting a novel role for LC8 in regulating HPIV3 P aggregation during infection. Finally, extensive sequence analysis using our LC8Pred algorithm demonstrates how certain viruses within Mononegavirales also possessed putative LC8 binding sites within their respective phosphoproteins.

In conclusion, we showed that both HPIV3 P and EBOV VP35 interact with LC8, though in different ways, suggestive of different functions in the context of infection. Future efforts characterizing the role of these interactions in an infection model are needed. Our study also indicates that LC8 can interact with pre-formed tetramers, not limiting its range to commonly reported dimers as previously reported in the literature. We showed that other related phosphoproteins from diverse families within Mononegavirales entail putative LC8 binding motifs that may have evolved independently. It remains to be seen why only a limited number of phosphoproteins entail LC8 binding anchors and the roles LC8 might play at the host–pathogen interface. Addressing this question will be a step toward better understanding of virus–host adaptations and may inform novel therapeutics against a broad range of viruses.

## Data Availability

Not applicable.

## Appendix A

**Table A1:** Virus abbreviations used in Figure 6C.

**Rhabdoviridae**	VSIV	Vesicular Stomatitis Indiana Virus	**Paramyxoviridae**	PIV5	Parainfluenza Virus 5
	VSNJV	Vesicular Stomatitis New Jersey Virus		HPIV2	Human Parainfluenza Virus 2
	VSAV	Vesicular Stomatitis Alagoas Virus		MapV	Mapuera Virus
	IsfV	Isfahan Virus		PPIV	Porcine Rubulavirus
	ChaV	Chandipura Virus		MAV	Menangle Virus
	PFV	Pike Fry Rhabdovirus		TioV	Tioman Virus
	SVV	Spring Viraemia of Carp Virus		TuhV	Tuhoko Virus 1
	TUPV	Tupaia Virus		HenV	Hendra Virus
	TBGV	Tibrogargan Virus		NipV	Nipah Virus
	BEFV	Bovine Ephemeral Fever Virus		CedV	Cedar Henipavirus
	OboV	Obodhiang Virus		CDV	Canine Distemper Virus
	ARV	Adelaide River Virus		PDV	Phocine Distemper Virus
	IrkV	Irkut Virus		RinV	Rinderpest Virus
	WCBV	West Caucasian Bat		HRSV	Human Respiratory Syncytial Virus
	EBLV2	European Bat Virus 2		HPIV3	Human Parainfluenza Virus 3
	MokV	Mokola Virus		MPIV	Murine Parainfluenza Virus
	LBV	Lagos Bat Virus		BPIV	Bovine Parainfluenza Virus
	RabV	Rabies Virus		HMPV	Human Metapneumovirus
	DUVV	Duvenhage Virus	**Filoviridae**	EbZV	Zaire Ebola Virus
	ABLV	Australian Bat Virus		TFEV	Tai Forest Virus
	SBLV	Shimoni Virus		BunV	Bundibugyo Virus
	AraV	Aravan Virus		MenV	Mengla Virus
	BBV	Bokeloh Bat Virus		EbSV	Sudan Ebola Virus
	SBV	Santa Barbara Virus		ResV	Reston Virus
				LLOV	Lloviu Virus
				MarV	Marburg Virus
